# Genome-wide analysis of SET domain genes and the function of *GhSDG51* during salt stress in upland cotton (*Gossypium hirsutum* L.)

**DOI:** 10.1186/s12870-023-04657-2

**Published:** 2023-12-19

**Authors:** Hongliang Jian, Fei Wei, Pengyun Chen, Tingli Hu, Xiaolan Lv, Bingqin Wang, Hantao Wang, Xiaohao Guo, Liang Ma, Jianhua Lu, Xiaokang Fu, Hengling Wei, Shuxun Yu

**Affiliations:** 1 National Key Laboratory of Cotton Bio-breeding and Integrated Utilization, Institute of Cotton Research of Chinese Academy of Agricultural Sciences, Anyang, Henan 455000 China; 2 Zhucheng Agricultural Technology Promotion Center, Zhucheng, Shandong 262200 China

**Keywords:** *Gossypium*, SET domain-containing, WGCNA, Salt stress, VIGS

## Abstract

**Background:**

Cotton, being extensively cultivated, holds immense economic significance as one of the most prominent crops globally. The SET (Su(var), E, and Trithorax) domain-containing protein is of significant importance in plant development, growth, and response to abiotic stress by modifying the lysine methylation status of histone. However, the comprehensive identification of SET domain genes (SDG) have not been conducted in upland cotton (*Gossypium hirsutum* L.).

**Results:**

A total of 229 SDGs were identified in four *Gossypium* species, including *G. arboretum*, *G. raimondii*, *G. hirsutum*, and *G. barbadense*. These genes could distinctly be divided into eight groups. The analysis of gene structure and protein motif revealed a high degree of conservation among the SDGs within the same group. Collinearity analysis suggested that the *SDGs* of *Gossypium* species and most of the other selected plants were mainly expanded by dispersed duplication events and whole genome duplication (WGD) events. The allopolyploidization event also has a significant impact on the expansion of *SDGs* in tetraploid *Gossypium* species. Furthermore, the characteristics of these genes have been relatively conserved during the evolution. *Cis*-element analysis revealed that *GhSDGs* play a role in resistance to abiotic stresses and growth development. Furthermore, the qRT-PCR results have indicated the ability of *GhSDGs* to respond to salt stress. Co-expression analysis revealed that *GhSDG51* might co-express with genes associated with salt stress. In addition, the silencing of *GhSDG51* in cotton by the virus-induced gene silencing (VIGS) method suggested a potential positive regulatory role of *GhSDG51* in salt stress.

**Conclusions:**

The results of this study comprehensively analyze the SDGs in cotton and provide a basis for understanding the biological role of SDGs in the stress resistance in upland cotton.

**Supplementary Information:**

The online version contains supplementary material available at 10.1186/s12870-023-04657-2.

## Background

As an important role in the process of histone methylation, the SET domain gene family is widely distributed in eukaryotes. The name of SET was taken from the three regulatory factors containing the SET domain, including *Su (var) 3–9* [1], *Enhancer of zeste* [2], and *Triohoarx* [3], which were first found in *Drosophila melanogaster*. SET domain gene (SDG) family could offer the activity of histone methyltransferase in the plant, which regulates plant histone lysine methylation. The SDG proteins are a specific catalytic site of histone lysine methyltransferase and are highly conserved in evolution [4].

The SDG family plays significant roles in the regulation of various biological processes, such as plant growth and development [5, 6]. The first reported SDGs in plants were the *CLF* (CURLYLEAF) and *MEA* (MEDEA) genes in *Arabidopsis* [7, 8], which could stably repress the floral homeotic gene and encoded a protein with homology to the product of the polycomb-group gene, *enhancer of zeste*, ensuring the stable inheritance of expression patterns through cell division and regulating the control of cell proliferation. In *Arabidopsis*, *TRITHORAX1* (*ATX1,* named *SDG27* in this study) has been shown to regulate floral organ development. *AtATX1* directly binds the active *FLC* locus before flowering, and this interaction is released upon the transition to flowering [9]. Meanwhile, *AtSDG40* affects flowering time by epigenetically modulated *FLC* expression. The decreased levels of H3K4me3 and increased H3K27me3 in *FLC* chromatin led to earlier flowering in *AtSDG40* mutants [10]. In rice, *OsSDG701* encoded an H3K4-methyltransferase that enhanced the expression of rice *Hd3a* and *RFT1* florigen, and promoted rice flowering under either long-day or short-day conditions [11].

In addition, SDG proteins have also been found to participate in response to external cues, including both abiotic and biotic stresses [12]. The chromatin modifier *AtATX1* participated in drought signal transduction in *Arabidopsis* [13]. At the same time, *AtATX1* affected the genes regulating the ABA pathway by up-regulating the expression of *NCED3* to improve drought stress tolerance [14]. Low temperature significantly affected the expression of *DcSUVH5a*, *DcATXR5a*, and *DcSUVR14a*, and the expression of *DcASHR3*, *DcSUVR3*, *DcATXR4*, *DcATXR5b*, and *DcSDG49* genes were affected by drought stress in orchids [15]. In *Arabidopsis*, the histone methyltransferase *AtSDG8* defends against fungal pathogens in plants by regulating a subset of genes in the jasmonic acid (JA) and ethylene signaling pathways [16]. These findings indicate that the *SDGs* could alter the histone methylation signal and affect the growth and adaptation of plants.

The identification and functional analysis of SDG gene family members has been deployed in many plant species, including *Arabidopsis thaliana* [17], *Oryza sativa* [18], *Zea mays* [19], *Gossypium raimondii* [20], *Brassica napus* [21], and *Triticum aestivum* [22] with 49, 43, 43, 52, 122, and 166 members, respectively. However, the study on *SDG* genes in upland cotton is still limited. In this study, a comprehensive identification of SDG proteins was conducted in four Gossypium species. This involved analyzing various aspects such as the analysis of the phylogenetic tree, chromosomal distribution, evolutionary, gene structure, domain organization, and *cis*-elements. In addition, the expression of *SDG* genes during plant growth and development and the response to various abiotic stresses were investigated. We further performed the qRT-PCR assays to verify the *GhSDGs* in response to abiotic stresses and hormonal treatments. In addition, the WGNCA analysis showed that *GhSDG51* co-expressed with many genes that are associated with salt stress. The further VIGS assays showed that the silencing of *GhSDG51* could reduce the salt stress tolerance in cotton plants compared with the control. Taken together, this research will provide valuable insights for future investigations on the SDG protein in cotton.

## Results

### Identification of SDG family members

By scanning the SET domain, a total of 40, 40, 75, and 74 SDG genes were identified in *G. arboreum*, *G. raimondii*, *G. barbadense*, and *G. hirsutum*, respectively. These genes were subsequently named as *GaSDG1*–*40*, *GrSDG1*–40, *GbSDG1*–*75*, and *GhSDG1–74* according to their chromosomal location. All of the members in the SDG family contained one SET domain, along with several other types of domains such as SAD, AWS, PWWP, MYDN finger, etc. (Fig. S1). The physical and chemical properties of the members of the SDG family members, including protein length, protein molecular weight (MWs), isoelectric point (pIs), and protein hydrophilicity and hydrophobicity were analyzed (Table S1). The distribution of values of the above physical and chemical characteristics in allotetraploid cotton and their diploid progenitors were approximately equal to each other (Fig. S2).

### Phylogenetic analysis and structure of SDG family members

**Fig. 1:**
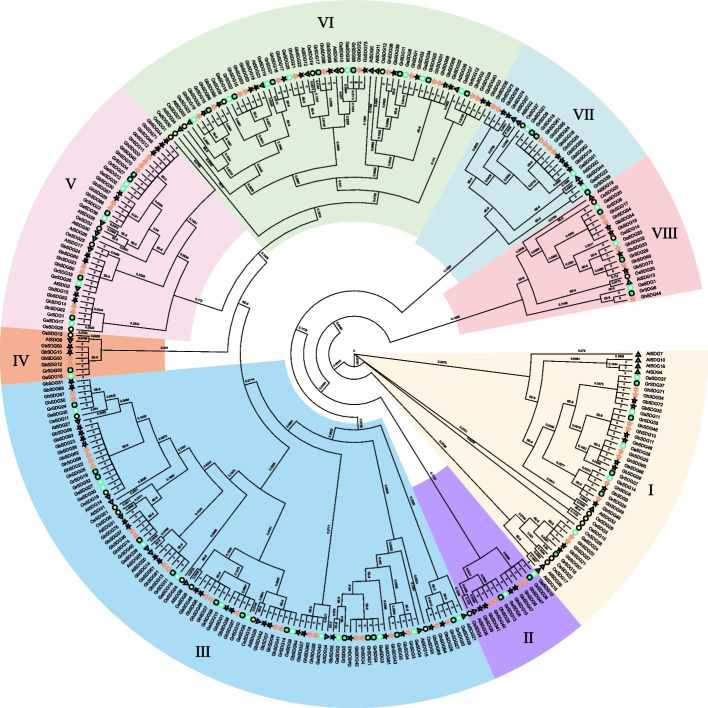
Phylogenetic tree of SDG proteins from six plant species. A phylogenetic tree was constructed using FastTree based on 297 SDG proteins obtained from *Arabidopsis thaliana*, *Oryza sativa*, *Gossypium hirsutum*, *Gossypium barbadense*, *Gossypium arboreum*, and *Gossypium raimondii*. The SDG proteins of the six species are denoted by distinct symbols and colors, while the eight subfamilies are distinguished by varying background colors

Combined with the identified SDG member in *Arabidopsis thaliana* and *Oryza sativa*, a total of 297 proteins were chosen to generate a phylogenetic tree (Fig. 1). As clearly shown in the phylogenetic tree, the SDG proteins can be divided into eight distinct subfamilies, namely subfamily I ~ VII. Each subfamily contains multiple SDG members from *Gossypium* species and at least one *AtSDG* member, indicating that there is no *Gossypium*-specific subfamily was found. Among the above subfamilies, the subfamily III contained the most members (30.97%), whereas the IV only contained seven members (2.35%). The member of each subfamily from *G. hirsutum* and *G. barbadense* was almost twice that of those from the diploid cotton.

**Fig. 2:**
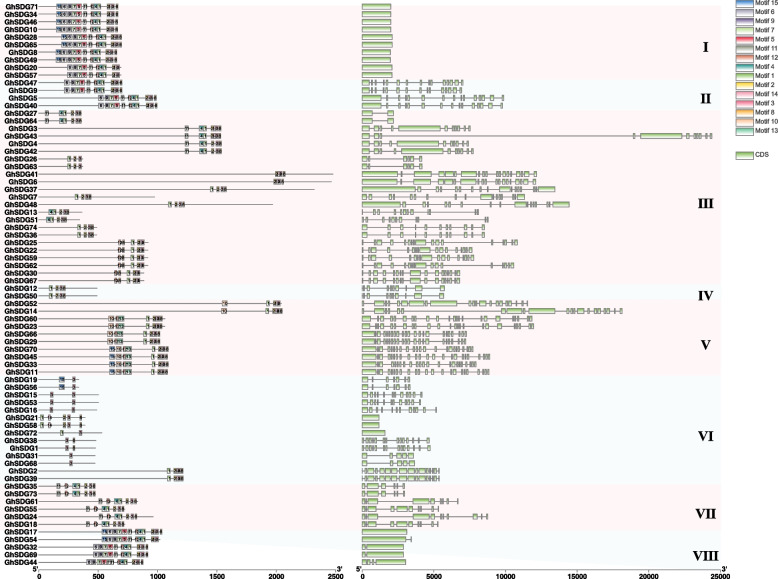
Conserved motifs and exon–intron structure analysis of *GhSDG* genes. **A** Conserved motifs of *GhSDG* proteins, the different motifs represented by boxes with different colors; **B** Exon–intron structures of *GhSDG* genes, the exons and introns are represented by green boxes and black lines, respectively. The scale at the bottom is used to infer the length of proteins and genes

To explore the structure of SDG members, a total of 15 motifs were identified (Fig. 2). These motifs performed different distributed patterns within each subgroup. The motif 3 (HIGLFAKRDIPAGEELTYDYG, identified as a part of SET domain) was detected in all of the subfamilies, and the motif 10 (WTTERCAVCRWVEDWDYNKIJICNRCQIAVHQECYGARNVRD, identified as a part of PHD-finger domain) and motif 13 (WVHVTCAWFRPEVSFADDEKMEPALGILRIPSDSFVKICVICKQIHGSCT, identified as a part of PHD-zinc-finger like domain) belonged exclusively to subfamily V (Table S2). The motif 11 was only detected in subfamily III and VII. On the other hand, the gene structure (exon-intron structure) of the *GhSDGs* exhibited significant variations across different subfamilies. All of the members in subfamily I displayed the mono-exon structure, while the members in subfamily V consisted of 21 ~ 24 exons. These results suggest that the gene structure and protein architecture of *GhSDGs* are conserved within each specific subfamily, implying that they may possess functions, and are consistent with the classification of the phylogenetic analysis.

### Gene duplication and selection pressure analysis of SDG members

**Fig. 3:**
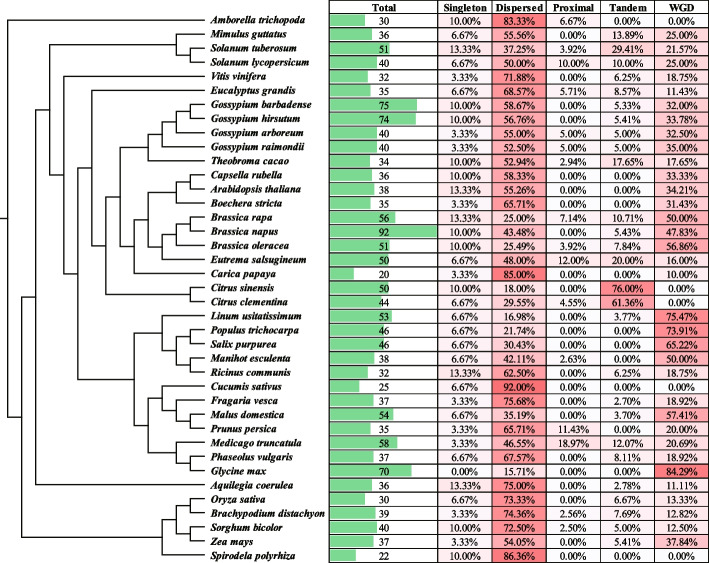
The phylogeny of 39 plants used in this study and the duplication events analysis of identified SDG proteins. The right side shows the amount and the duplication types of SDG protein detected in each plant. The order of tree branches depicted on the left side was retrieved from the Timetree database (http://www.timetree.org/)

To investigate the expansion pattern of *SDG* genes, we conducted a further analysis to identify the duplication pattern. Multiple types of duplication events were detected in cotton’s *SDG* genes. The expansion of the *SDG* genes in *Gossypium* species was primarily driven by the dispersed duplication event, with the subsequent whole genome duplication (WGD) event also playing a significant role (Fig. 3 and Table S3). These results provide an explanation for the observation that the number of *SDG* genes in diploid *Gossypium* species is only 1.18 times higher than in *T.cacao*, despite *Gossypium* species undergoing an additional round of WGD event compared to *T.cacao* (Fig. 3). A comprehensive search was conducted in an additional 35 plant species, resulting in the identification of a total of 1689 *SDG* genes across 39 species (Fig. 3). We found that *SDGs* were widespread in monocotyledonous and dicotyledonous plants. Among these species, the *SDG* genes in most species were mainly expanded by dispersed duplication, the proportion of this duplication varying from 46.5% ~ 92% (Fig. 3). However, the WGD and tandem duplication events were the major force in expanding the *SDG* genes in some cases, the 84.3 and 76% of *SDG* in *Glycine max* and *Citrus sinensis* were expanded by WGD and tandem duplication events, respectively (Fig. 3). These results indicated that the expansion of *SDG* genes in the majority of species is likely to be driven by dispersed events. However, in a few instances, the expansion of *SDG* genes may also be expanded by WGD and tandem duplication events.

All the Ka/Ks ratios of gene pairs of *SDG* genes in *Gossypium* species, resulting from various duplication events, were found to be lower than 1. This observation suggests the *SDG* genes might experience strong purifying selection pressure during evolution and suggests that the protein functions may be conserved after the expansion (Table S4).

**Fig. 4:**
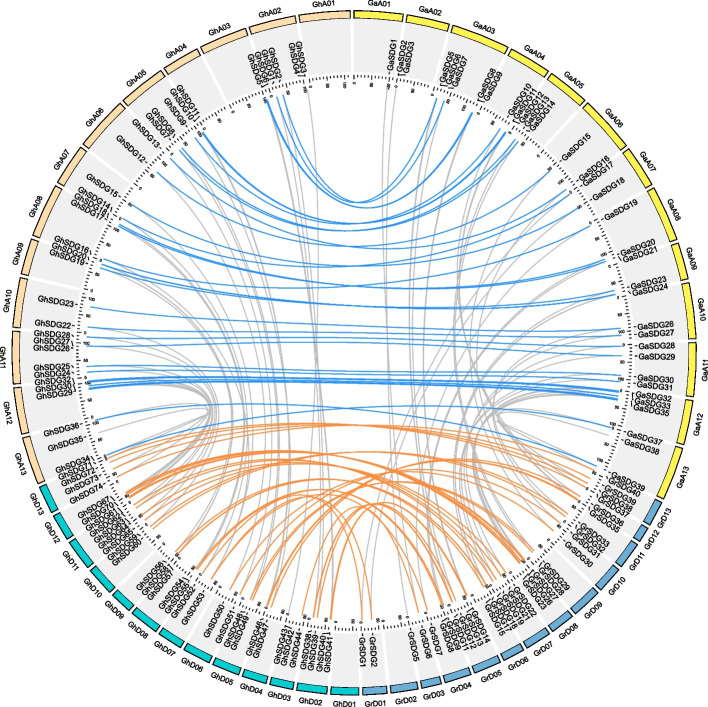
Chromosome distribution and collinearity of duplicated gene pairs of SDG genes. Chromosomes of the *Gossypium arboreum, Gossypium raimondii,* and the A_t_ and D_t_ subgenomes of *Gossypium hirsutum* are represented by differently colored boxes. Collinear gene pairs between *GhSDG* genes and the SDG genes of the diploid species are represented by blue and orange lines

The allopolyploidization event has been found to have a significant impact on the expansion of *SDG* genes in cotton. The number of members in the tetraploid cotton species was nearly double that of the diploid cotton species, whereas some SDG members might have been lost during the evolution. The *SDG* genes of the four *Gossypium* species exhibited an uneven distribution across nearly all of the chromosomes (Fig. 4 and S3; Table S1). For *G. arboreum*, the *SDG* genes were unevenly distributed on each chromosome, while the *SDG* genes of *G. raimondii* were distributed on 12 chromosomes except D02. In both the *G. hirsutum* and *G. barbadense*, *SDG* genes were absent on A01, A04, and D01. The distribution of *SDG* genes in the *G. arboreum* and At subgenome of tetraploid cotton species exhibited a high level of consistency. However, the distribution of *G. raimondii* showed slight differences compared to the Dt subgenome of tetraploid cotton species (Fig. 4 and S3). We also performed a collinearity analysis of the *SDG* genes between the *Gossypium* species and *Arabidopsis*. The detected collinearity gene pairs account for less than half of the *SDG* genes, indicating that these genes may have been conserved from their common ancestor and the rest of the genes might be duplicated in different evolutionary events (Fig. S4).

### *Cis*-elements analysis of *GhSDGs*

The *cis*-element related to the stress response, light response, growth and development were detected in the promoter region (2000 bp upstream of the start codon) of *GhSDGs*. The results displayed that the MYC, MYB, and STRE elements were enriched in the promoter of *GhSDGs*, indicating that these *GhSDGs* are likely to play a crucial role in the response to environmental stress (Fig. S5). Therefore, we speculated that *GhSDGs* might play a vital role in regulating the defense against the external environment.

### The expression pattern of *GhSDGs*

According to the analysis of the RNA-seq data, the *GhSDGs* exhibited distinct expression patterns across various tissues. The *GhSDG34*, *20*, *8*, and *49* were expressed relatively highly in all tissues, whereas the *GhSDG43*, *14*, *35*, and *60* were rarely expressed in all tissues (Fig. S6). *GhSDG13* exhibits a specific and highly expressed pattern in both the leaf and root, while *GhSDG72* shows exclusive high expression specifically in the root. These results indicate that *GhSDGs* might play different roles in the growth and development in upland cotton.

**Fig. 5:**
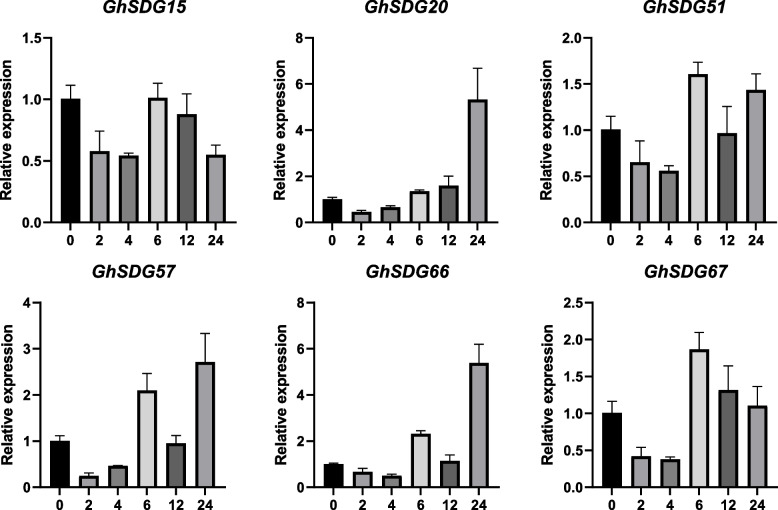
The relative expression of *GhSDGs* under the salt treatment. *GhActin7* was used as an internal reference. The primers used are listed in Table S5. Error bars represent the standard deviation of three independent biological replicates

The biological function of a specific gene is closely related to the spatiotemporal expression of the transcript. Based on the results of RNA-seq data (Fig. S7), we chose 6 genes to performed qRT–PCR analysis to investigate their response under the NaCl treatments. The results showed that the under the salt treatment, the expression of *GhSDGs* were down-regulated after the salt treatment at 2 h and 4 h, while their expression rebound at the 6 h ~ 24 h. The expression of *GhSDG20* reached the peak at 24 h, while the expression of *GhSDG15* and *GhSDG67* continuous decreasing after 6 h. The expression of *GhSDG51*, *GhSDG57*, *GhSDG57*, and *GhSDG67* decreased at 12 h but increased at 24 h (Fig. 5). These results indicating that *GhSDGs* could respond to the salt stress.

### WGCNA network analysis predict *GhSDG51* related to salt stress

**Fig. 6:**
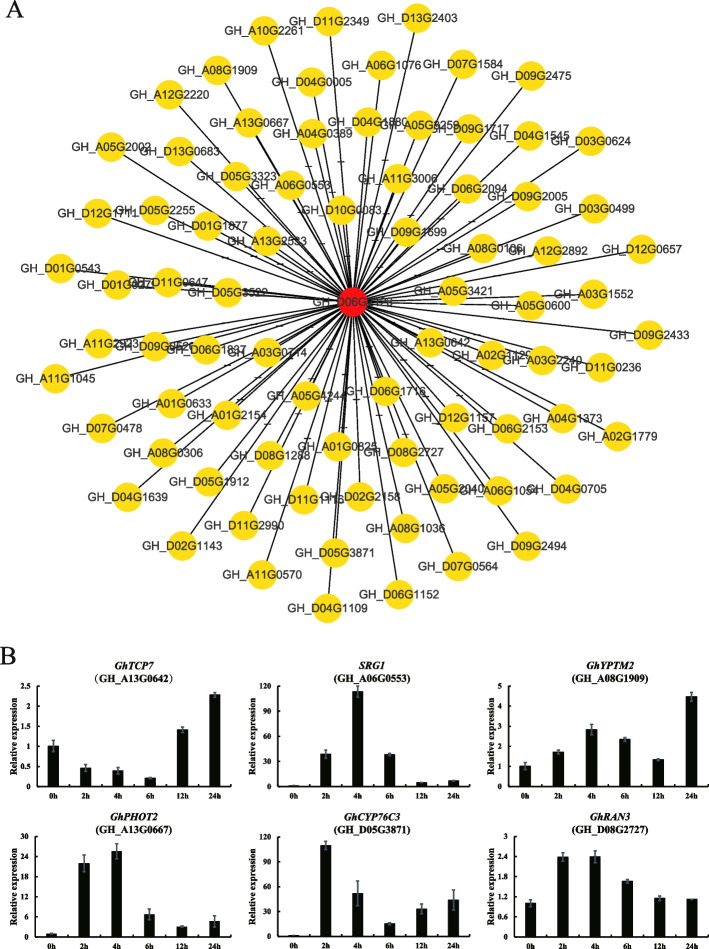
Co-expression network and qRT-PCR analysis. **A** The co-expression network of *GhSDG51* in salt stress. The information of genes displayed in the network were listed in Table S6. The raw RNA-seq data (seedlings under the salt treatment for 0, 3, 6, 9, 12, and 24 h) was retrieved from a previous study (accession number: PRJNA490626). **B** The relative expression of the genes that co-expressed with *GhSDG51*. *GhActin7* was used as an internal reference. The primers used are listed in Table S5. Error bars represent the standard deviation of three independent biological replicates

We have additionally discovered that GhSDG51 acted as a hub gene within a weighted gene co-expression network analysis (WGCNA) network that is associated with salt stress. In this network, *GhSDG51* exhibited co-expression with several transcription factors, including *GhSRG1*, *GhYPTM2*, *GhPHOT2, GhCYP76C3*, *GhRAN3* and *GhTCP7* (Fig. 6). We further found that these genes could respond to salt stress by qRT-PCR assays. The expression levels of *GhYPTM2*, *GhPHOT2*, *GhCYP76C3* and *GhRAN3* showed an overall upward trend after salt stress. The expression of *GhSRG1* increased at 2 h, 4 h, and 6 h after salt treatment, and the expression of *GhTCP7* increased at 12 h and 24 h after salt treatment. Therefore, it was hypothesized that *GhSDG51* might play a role in the response to salt stress.

### Silence *GhSDG51* reduces tolerance to salt stress

**Fig. 7:**
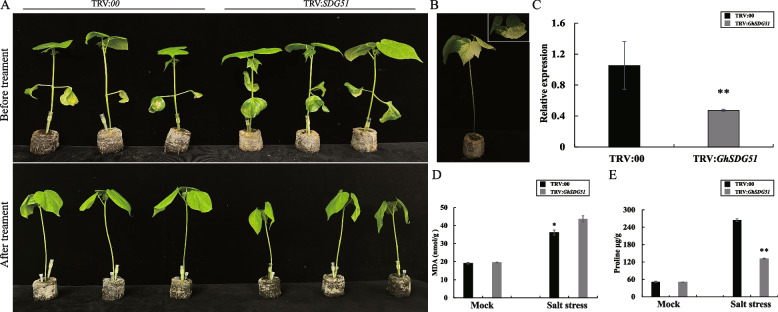
Silencing *GhSDG51* reduced tolerance to salt stress. **A** Phenotype of empty control (TRV: 00) and VIGS plants (TRV: *GhSGD51*) before and under 400 mM NaCl treatment (TRV: *GhSGD51*); **B** Positive control (TRV: *CLA1*); **C** The expression level of *GhSDG51* in blank control and VIGS plants; **D** The malonaldehyde (MDA) contents of blank control and VIGS plants; **E** The proline (Pro) contents of blank control and VIGS plants. The data measurement was tested with a one-way analysis of variance (ANOVA) using the student’s t-test. ** and * indicate statistical significance at the 0.01 and 0.05 probability levels, respectively

To further explore the function of *GhSDG51* under salt stress, we silenced the *GhSDG51*gene in cotton by VIGS assay. After approximately 2 weeks following the injection, the TRV: *GhCLA1* plants exhibited an albino phenotype (Fig. 7B), indicating that the VIGS experiment was successful. Furthermore, the qRT-PCR experiment revealed a significant decrease in the expression level of *GhSDG51* in TRV: *GhSDG51* plants compared to TRV: 00 plants (Fig. 7C), indicating that this gene expression was successfully silenced. After the salt treatment with 400 mM NaCl for 2 days, it was observed that the *GhSDG51*-silenced plants exhibited noticeable wilting in comparison to the control group (Fig. 7A). To further investigate the impacts of plant physiological and biochemical characteristics, the content of proline (Pro) and malondialdehyde (MDA) were measured in TRV: *GhSDG51* and TRV: 00 plants. Before treatment, the content of MDA and proline in TRV: *GhSDG51* and TRV: 00 plants exhibited similarity. After the treatment, the content of MDA was found to be significantly higher in TRV: *GhSDG51* plants compared to TRV: 00 plants. Conversely, the content of proline in TRV: *GhSDG51* was significantly lower than that in TRV: 00 plants (Fig. 7D, E). These results indicated that the salinity tolerance of cotton plants was reduced following the silencing of *GhSDG51*. Moreover, it is proposed that *GhSDG51* may play a crucial role in positively regulating the response of cotton to salt stress.

## Discussion

Cotton, a significant economic crop known for its inherent abiotic resistance, is extensively cultivated worldwide. Abiotic stresses, such as salinity stress, have a significant impact on the growth of cotton and have led to limitations in its cultivation worldwide. The SET domain-containing proteins (SDGs) are known histone lysine methyltransferases and participate in several processes, including responding to both biotic and abiotic processes [23, 24]. Here, we conducted a comprehensive genome-wide investigation of SDGs in *G.hirsutum* and explored their potential in salt stress.

This study identified a total of 229 putative SDG genes from *G. arboreum*, *G. raimondii*, *G. barbadense*, and *G. hirsutum*. In both allotetraploid cotton species *G. hirsutum* and *G. barbadense*, the number of identified SDG genes was nearly equal to the combined number of SDG genes in diploid cotton varieties *G. arboreum* and *G. raimondii*. This trend was also observed within each subfamily. Further analysis of gene structure and protein sequences revealed that SDG gene members within each subfamily exhibited similar gene structures, sequence lengths, and motif distribution pattern. Therefore, our findings potentially support previous assertion that the allotetraploid *Gossypium* species are evolved and speciated by the hybridization of the diploid progenitors (Table S1, Fig. 1).

We further found that the dispersed duplication event is the main force in the expansion of the *SDG* genes in many plants, including the *Gossypium* species (52.5% ~ 58.67%), which is different from the duplication pattern on the whole-genome level (Table S3). In addition, compared with the diploid *Gossypium* species, most SDG genes in allotetraploid species were well conserved in the allotetraploidization events, a similar result was also found in the *Brassica* species in this study (Fig. 4, Fig. 3). Functional differentiation might occur in the duplicated genes, including partial or complete loss of the previous functions, gaining new functions, or maintaining the original functions [25]. And the selection pressure analysis showed that the *SDG* genes are under the purified selection during the multiple duplication events (Table S4). These results suggest that members of the SDG family may exhibit relatively conserved functions during the evolution.

Promoters contain *cis*-elements that are crucial for the specific expression and function of a gene during growth and development. Some critical stress and hormone-responsive *cis*-elements for the plant were found in the promoter region of almost all SDG genes (Fig. S5). Therefore, promoter analysis indicated that the SDG genes in cotton played roles in various stress responses. By performing the RT-PCR, many SDG genes were found to respond to the salt treatment, including *GhSDG51*. The WGCNA analysis further showed that *GhSDG51* might act as a hub gene in a co-expression network related to salt stress. This network includes *GhRAN3*, *GhYPTM2*, *GhSRG1*, *GhPHOT2*, *GhTCP7*, and *GhCYP76C3*, which could participate in the process of plant cell activity, signal transduction, and responding to multiple abiotic stresses [26–31]. Next, the qRT-PCR assays indicated the genes that co-expressed with *GhSDG51* were also up-regulated in salt stress (Fig. 6).

Based on the above results, we speculate that *GhSDG51* is a candidate gene that may play a role in salt stress. Due to factors such as the long time and low efficiency of the cotton transgene, we performed VIGS assay to silence of *GhSDG51* to study its functions because of the short experimental period, simple operation method, low cost, and high efficiency of this method. After salt treatment, the TRV: *GhSDG51* plants wilted more leaves compared to control plants (Fig. 7A). Additionally, after salt treatment, the TRV: *GhSDG51* plants possessed higher MDA, whereas the content of proline was lower than that in control plants (Fig. 7D, E). The proline regulates osmotic potential and scavenging ROS, the content of proline is positively correlated with the abiotic stress resistance of plants, including salt, drought, and high temperature stress [32, 33]. While MDA is the main product of membrane lipid peroxidation, and the content of MDA indirectly reflects the damage degree to plant cells [34]. Therefore, we conclude that *GhSDG51* could play a positive role in salt stress. However, the precise mechanism of the function of *GhSDG51* requires further study.

## Conclusion

In this study, 229 cotton SDG genes were identified in *G. arboreum*, *G. raimondii*, *G. hirsutum*, and *G. barbadense*, and they could be divided into eight subfamilies. The dispersed duplication and allopolyploidization events played a vital role in the expansion of *GhSDGs*. The analysis of *cis*-elements and the qRT-PCR experiments demonstrated that *GhSDGs* might respond to abiotic stresses. The WGCNA analysis further showed *GhSDG51* plays as a hub gene in a co-expression network. Finally, by performing the VIGS assay and the measurement of physiological traits, the *GhSDG51* might play a positive role in salt tolerance. This study laid a foundation for further understanding of SDG proteins in cotton, and provided functional candidate genes related to salt stress.

## Material and methods

### Identification and characterization of SDG proteins

The HMMER software (version 3.3.2) was performed to screen the SDG protein sequences containing SET domain (PF00856) with default parameters in 39 selected plant species, the detailed information on these species is listed in Table S7 [35]. To further confirm that the candidate SDGs contain the SET domain, we employed the InterProScan program (version 5.51RC1–84.0) with the SMART database (SM00317) and PFAM database (PF00856) [36]. The protein properties of the SDGs were calculated by the ProtParam module in Biopython [37].

### Phylogenetic analysis of SDG proteins

The SDG protein sequences retrieved from the *A. thaliana*, *O. sativa*, and four *Gossypium* species were aligned by performing the MAFFT software (version 7.310) [38]. The gaps in the alignment result were further removed by BMGE software [39]. The phylogenetic tree was constructed by the aligned protein sequences by FastTree (version 2.1.11) [40]. The visualization of the phylogenetic tree was generated by evolview (version 3.0) [41].

### Gene structure, and motif analysis of *GhSDGs*

The information on gene structure (exon/intron) was retrieved from the genomic annotation (gtf/gff files). The SDG protein sequences were submitted to the MEME website to detect the motifs [42]. The PlantCARE website was used to detect the *cis*-elements in the promoter (2000 bp upstream genomic DNA sequences) of SDG genes [43]. The results were visualized in TBtools (version 0.1098765) software [44].

### Analysis of gene collinearity and duplication events

The duplication event was identified by the duplicate_gene_classifier module in MCScanX [45]. The collinearity of the SGD genes was calculated by MCScanX and Dupgen_finder [45, 46]. The gene collinearity and the chromosome location of SDGs were visualized in Circos software (version 0.69–9). The ratio of nonsynonymous rate and synonymous rate (Ka/Ks) was calculated by ParaAT pipeline and Kaks_calculator (version 2.0) [47, 48].

### Expression proflings and WGCNA analysis

The transcriptome data from a previous study were used in this study (Accessions: PRJNA490626) [49]. The analysis pipeline for generating FPKM (Fragments Per Kilobase of transcript per Million mapped reads) value and the analysis of WGCNA were described in our previous study [50].

### RNA extraction and qRT-PCR analysis

The seeds of upland cotton (TM-1) were cultured in seedling blocks at 24 / 20 °C for 16 / 8 h. Three-week-old seedlings were treated selected with 200 mM sodium chloride. Three biologically replicated of true leaves were collected at 0, 2, 4, 6, 12, and 24 h and frozen in liquid nitrogen at − 80 °C for further analysis. Total RNA was isolated using the RNA prep Pure Plant Kit (TIARGEN, Beijing, China) and treated with DNase-I to remove genomic DNA. The quality and purity of the RNA were measured by a NanoDrop 2000 spectrophotometer (Thermo Scientific, Massachusetts, USA), and 1 μg of RNA was used to synthesize first-strand cDNA with the Transcriptor First Strand cDNA Synthesis Kit and oligo-dT primers at 42 °C for 60 min, followed by 72 °C for 10 min. Real-time PCR was performed in a 7500 Fast Real-Time PCR system (Applied Biosystems, State of California, USA) using SYBR Green Master Mix. Three biological replicates were performed for each cDNA sample, and each reaction was prepared in a total volume of 20 μL containing 10 μL of SYBR Green PCR Master Mix, 0.4 μL of each primer, 2 μL (200 ng) of diluted cDNA template, and 7.2 μL of nuclease-free H_2_O. The qRT-PCR was performed using the PCR System from Applied Biosystems. The primers for the qRT-PCR are listed in Table S5. Results were analyzed with the 2^−∆∆Ct^ method [51].

### VIGS of *GhSDG51* in upland cotton

A specific fragment of *GhSDG51* (301–600) was cloned and used to construct a pTRV2:*GhSDG51* vector. Then, the recombinant vector plasmid was transformed into *Agrobacterium tumefaciens* LBA4404. The *Agrobacterium tumefaciens* carrying pYL156 (negative control, TRV: 00), pYL156-*GhSDG51* (TRV: *GhSDG51*), pYL156-*CLA1* (positive control, TRV: *CLA1*), and pYL192 (accessory vector) were cultured in 28 °C, 180 rpm shaker. When the OD600 of the bacterial liquid reached 1.8, the bacteria were resuspended to OD600 = 1.5 by using the buffer containing 10 mM MgCl_2_, 10 mM MES, and 200 μM acetosyringone. After being kept in the dark at 25 °C for 3 h, the Agrobacterium resuspended solution containing pTRV2: *GhSDG51*, pTRV2: 00, and pTRV2: *GhCLA1* was mixed with pYL192 in a 1:1 ratio [52]. The mixture was then injected into the cotyledons of TM-1 seedlings. Finally, the injected cotton plants were placed in a dark incubator and moved to the culture room for regular cultivation after 24 h. After growing for about 2 weeks, when the leaves of positive plants exhibited an albino phenotype, the silencing efficiency was subsequently determined through qRT-PCR experiments. The TRV: *GhSDG51* and TRV: 00 were irrigated with 400 mM NaCl to observe the phenotype. Cotton leaves were used to determine the content of MDA content and proline activity by using the malondialdehyde assay kit (Jiancheng, Nanjing, China) and proline (Pro) assay kit (Jiancheng, Nanjing, China), respectively.

### Supplementary Information

**Additional file 1.**

**Additional file 2.**

**Additional file 3.**

**Additional file 4.**

**Additional file 5.**

**Additional file 6.**

**Additional file 7.**

**Additional file 8.**

## Data Availability

Genome files of *Arabidopsis thaliana*, *Oryza sativa*, Gossypium species, and Brassica species were obtained from TAIR (https://www.arabidopsis.org/), Rice Genome Annotation Project (http://rice.uga.edu/), Cottongen (https://www.cottongen.org/), BRAD (https://brassicadb.org/) respectively. The genome files of the rest species mentioned in the article were retrieved from the Phytozome (https://phytozome-next.jgi.doe.gov/). The RNA seq data used in this study were retrieved from the NCBI Sequence Read Archive (SRA) database (https://www.ncbi.nlm.nih.gov/sra) under the accession code PRJNA490626.
