# Supplementary material for: Genome-wide analysis of SET domain genes and the function of GhSDG51 during salt stress in upland cotton (Gossypium hirsutum L.)
Source: BMC Plant Biol. 2023 Dec 19;23:653. doi: 10.1186/s12870-023-04657-2 (PMC10729455; doi:10.1186/s12870-023-04657-2)
Supplement: Supplementary file 1 — Additional file 1. [file 12870_2023_4657_MOESM1_ESM.docx]

**Supplementary information**


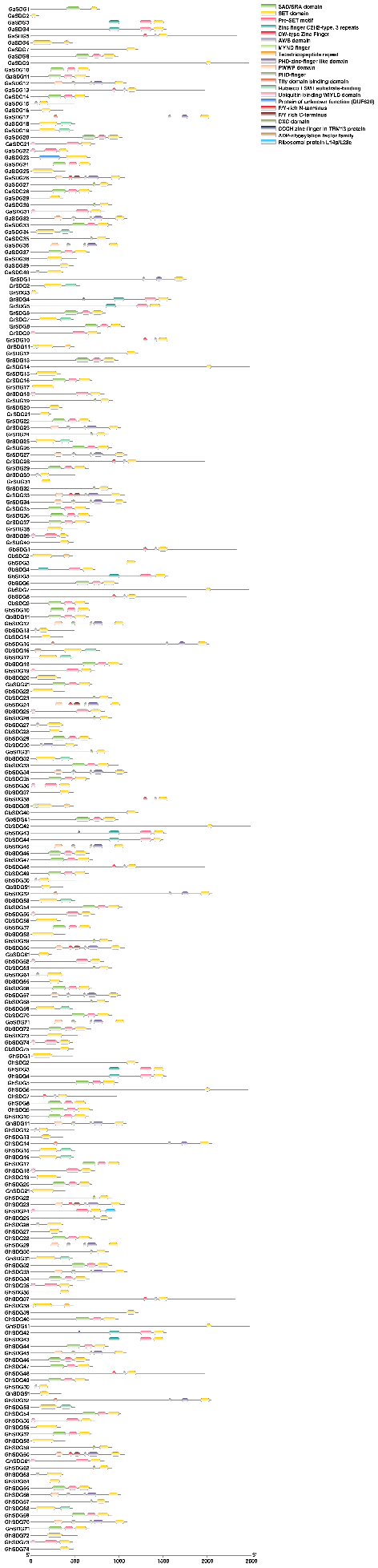


**Figure S1**. Domain analysis of SDG proteins. Conserved domains of SDG proteins were identified using Pfam. The different domain are represented by different colors.


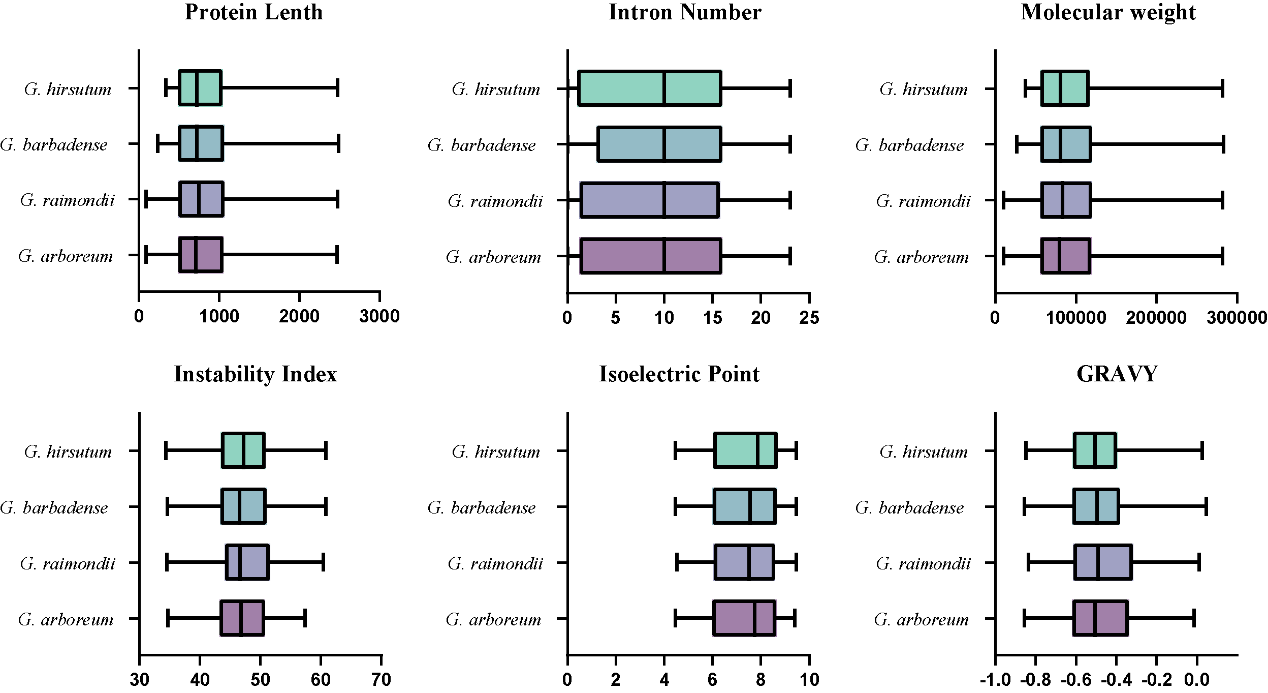


**Figure S2**. The physical and chemical parameters SDG proteins. The original data was list in Table S1.

**
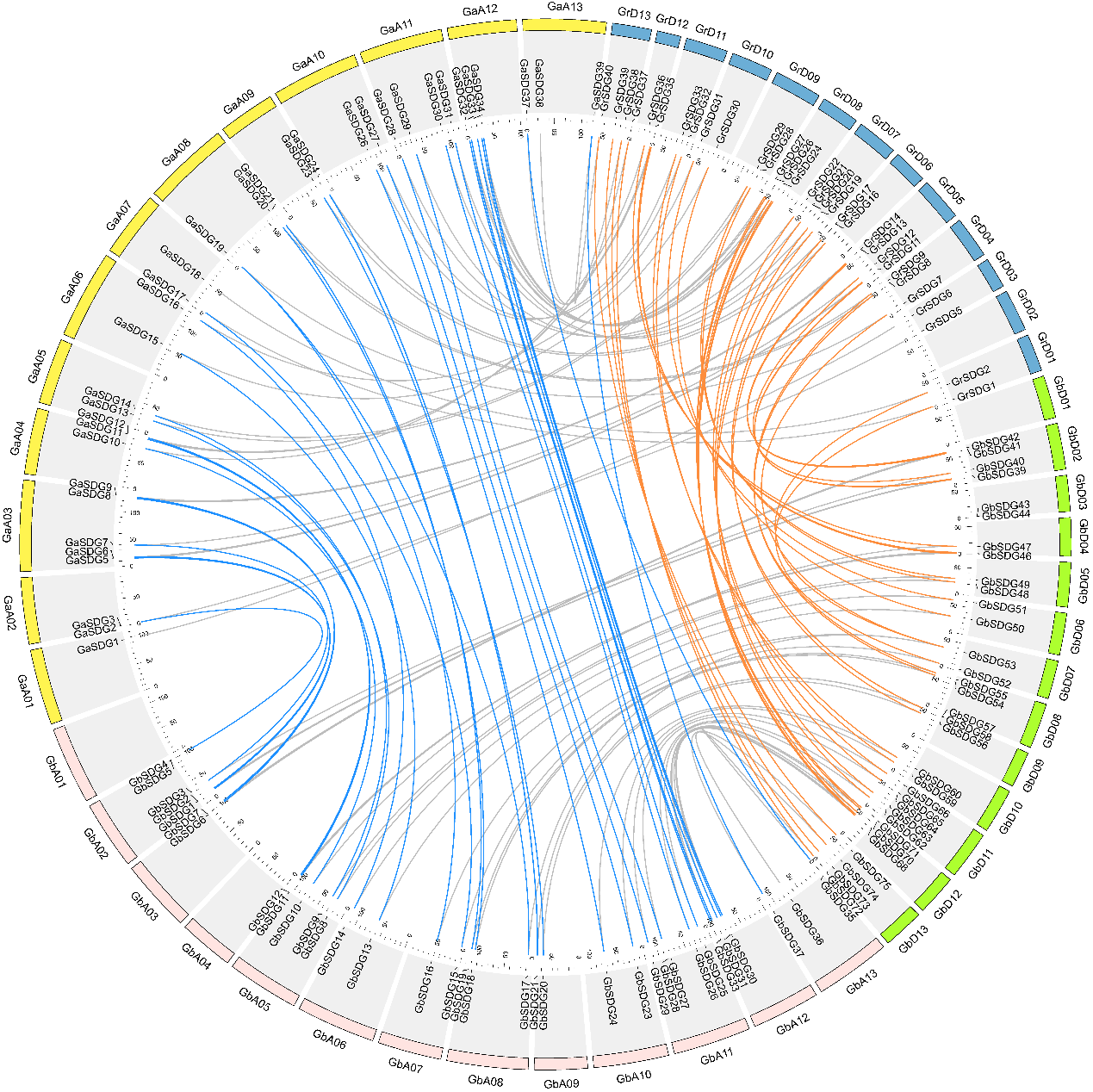
**

**Figure S3.** Chromosome distribution and collinearity of duplicated gene pairs of SDG genes. Chromosomes of the *Gossypium arboreum*, *Gossypium raimondii*, the At and Dt subgenomes of *Gossypium barbadense* are represented by differently colored boxes. Collinear gene pairs between *GbSDG* genes and the SDG genes of the diploid species are displayed by lines in different colors.


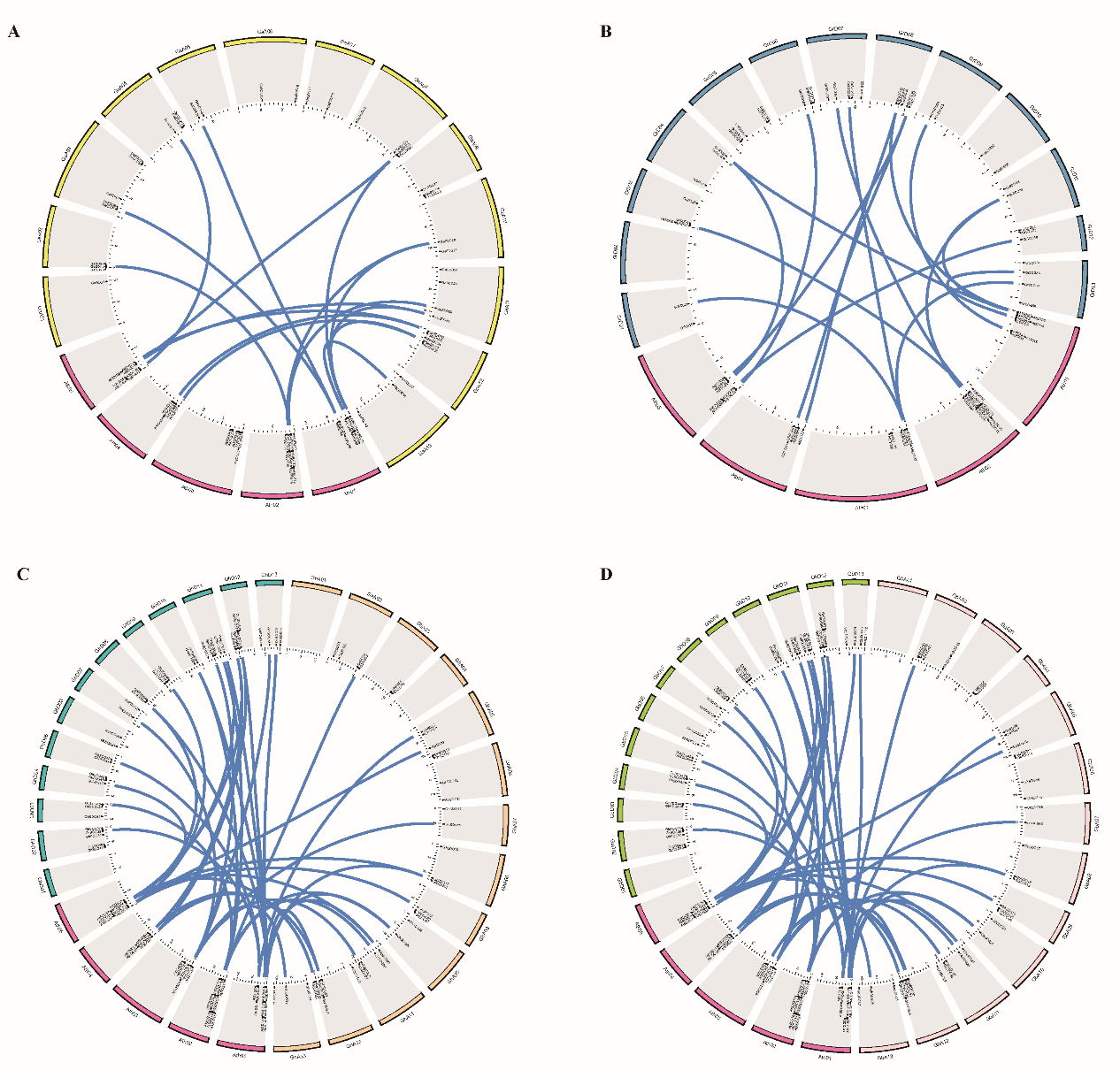


**Figure S4.** The collinearity analysis between *Gossypium* species and Arabidopsis. Chromosomes of the genome and subgenomes are represented by differently colored boxes. The gene pairs of *SDG* genes between *Gossypium* species and Arabidopsis were displayed in blue.

**
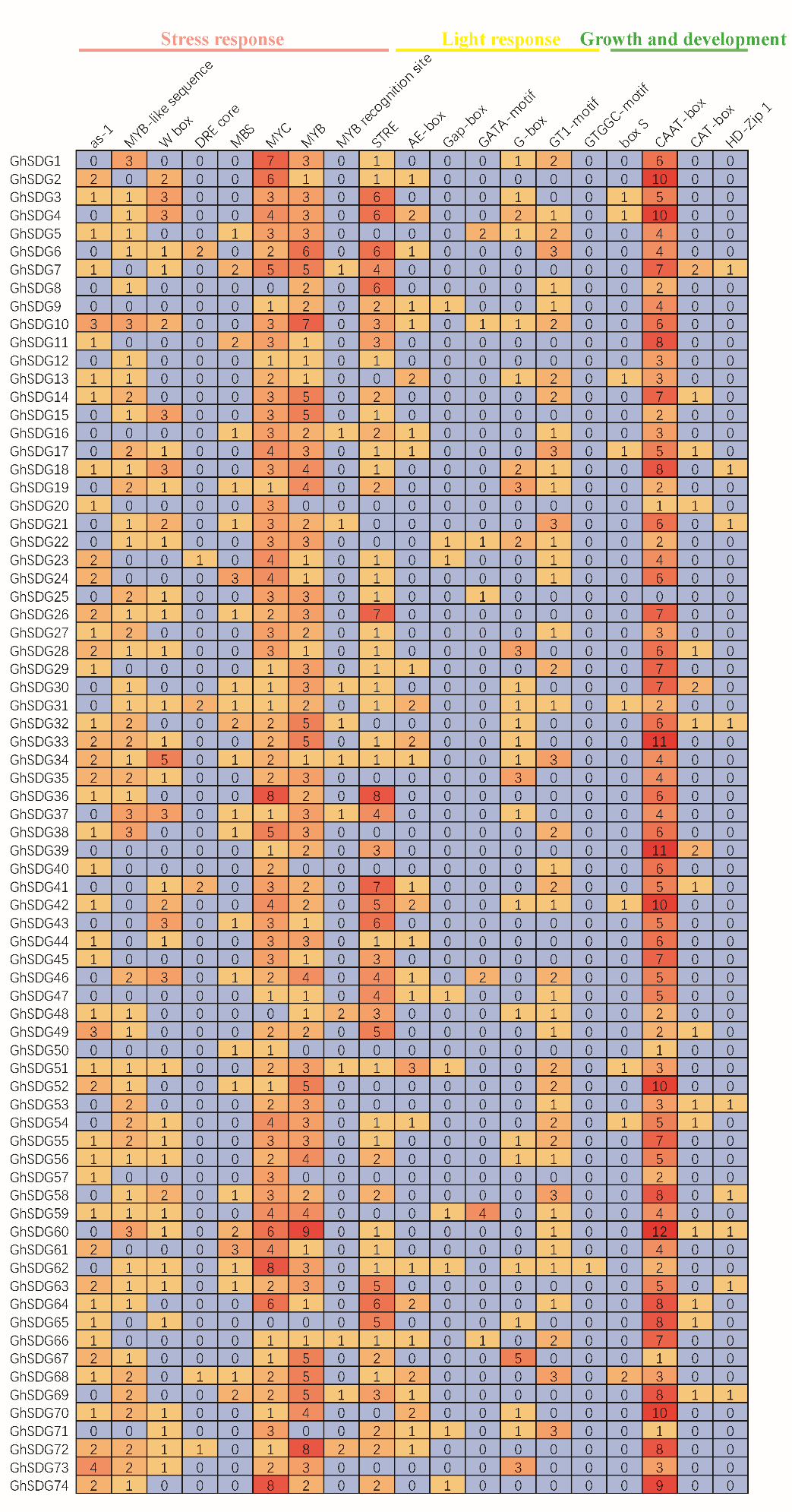
**

**Figure S5.** *Cis*-element analysis of the *GhSDG* gene’s promoter.

**
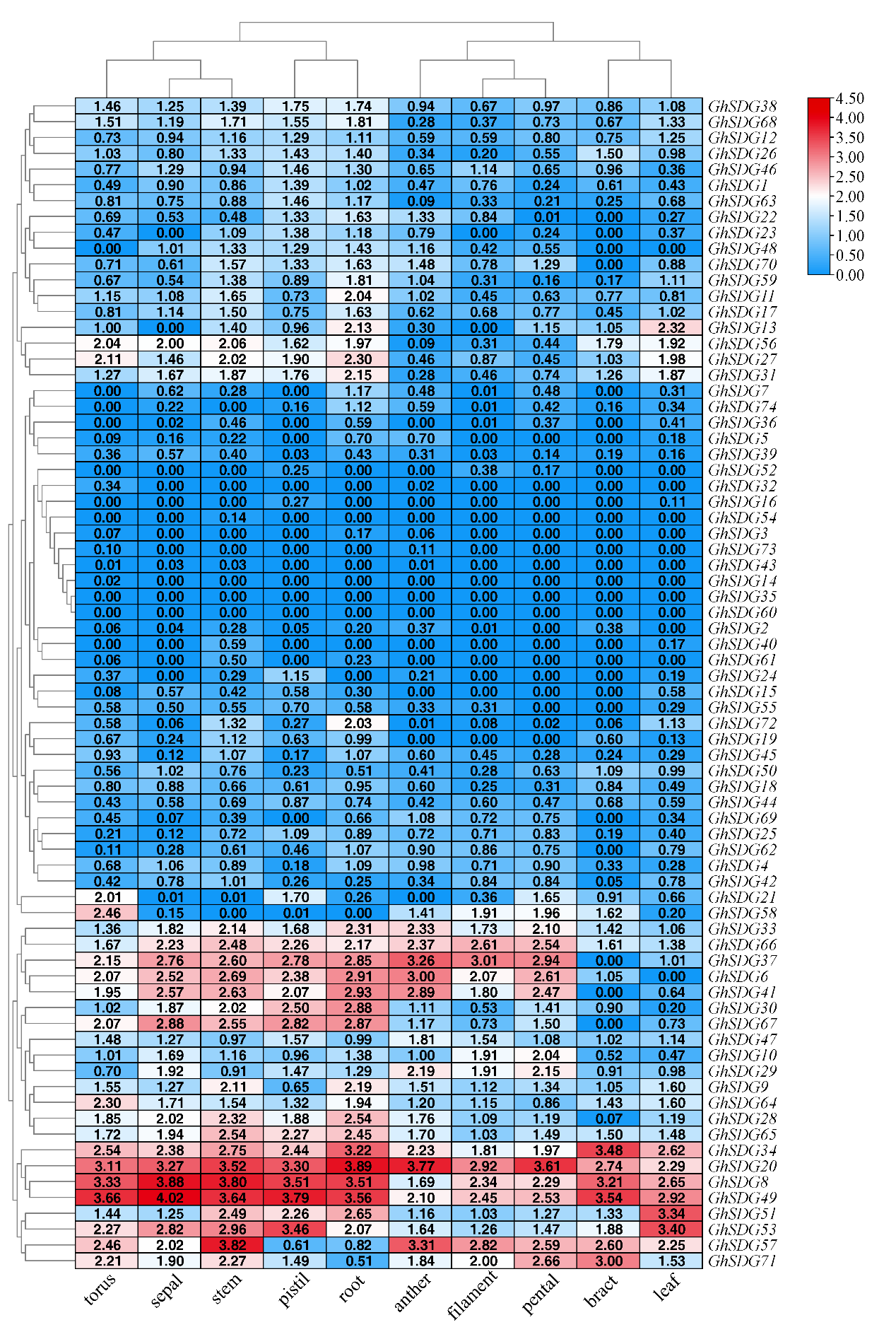
**

**Figure S6.** Expression patterns of *GhSDGs* in different tissues in *G. hirsutum.* The yellow indicates a high expression level and blue indicates a low expression level in the scale bar. The number displayed in each boxes represent log2 transformed FPKM values. The raw RNA-seq data was retrieved from a previous study (accession number: PRJNA490626).


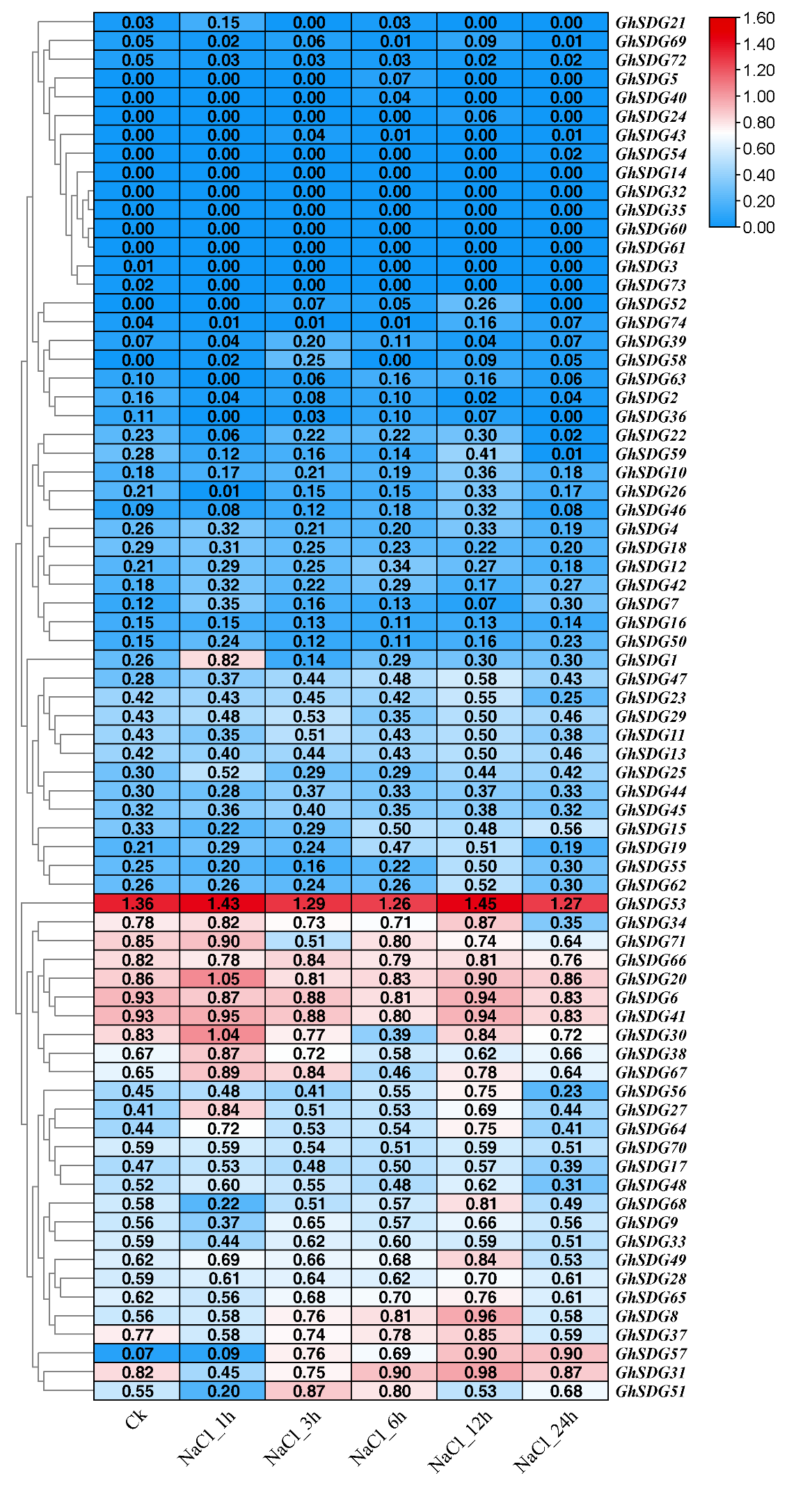


**Figure S7.** Expression patterns of *GhSDGs* under the salt treatment*.* The yellow indicates a high expression level and blue indicates a low expression level in the scale bar. The number displayed in each boxes represent log2 transformed FPKM values. The raw RNA-seq data was retrieved from a previous study (accession number: PRJNA490626).
